# Postoperative Load Bearing in Periprosthetic Femoral Fractures Around Hip Arthroplasty: A Survey Among Orthopedic Surgeons in the Netherlands

**DOI:** 10.7759/cureus.45122

**Published:** 2023-09-12

**Authors:** Maud A.M. Vesseur, Jetse Jelsma, Jasper Most, Yoeri F.L. Bemelmans, Martijn G.M. Schotanus, Raoul van Vugt, Bert Boonen

**Affiliations:** 1 Department of Orthopedic Surgery, Zuyderland Medical Center, Sittard-Geleen, NLD; 2 Department of Surgery, Zuyderland Medical Center, Sittard-Geleen, NLD

**Keywords:** trauma, survey, orthopedic surgery, permissive weight bearing, periprosthetic femoral fractures

## Abstract

Introduction: Permissive weight bearing (PWB) has relatively recently been implemented to optimize rapid clinical recovery and restoration of function in patients suffering lower extremity fractures. PWB shows outcome advantages in this patient category. Currently, there are no decisive recommendations on postoperative load-bearing management after surgically treated periprosthetic femoral fractures (PPFF) around hip arthroplasty. The objective is to investigate the current postoperative practice of weight-bearing instructions for patients with surgically treated PPFF, accounting for differences in types of periprosthetic fractures and treatment options among Dutch orthopedic surgeons.

Materials and methods: An online survey was distributed among the members of the hip and trauma working groups of the Dutch Orthopedic Association.

Results: The response rate was 13% (n=75). The main finding was that postoperative load bearing regimes in Vancouver A, B, and C PPFFs differed greatly among Dutch orthopedic surgeons, and there was no decisive guideline or consensus in postoperative load bearing regimes after surgically treated PPFF was used in the Netherlands.

Conclusion: In the absence of decisive guidelines or consensus, more research is needed to explore the efficacy of PWB after surgically treated PPFF.

## Introduction

In 1891, the first recognized hip replacement was performed [1]. Periprosthetic femoral fractures (PPFF) after total hip arthroplasty (THA) were first reported in 1954 [2]. The aging population results in a higher prevalence and incidence of osteoarthritis and, therefore, primary THA [3-4]. In addition, implant designs have improved, which increases their lifetime. Consequently, numbers of PPFF are also rising [4-5]. A wide range is observed in the probability of PPFF after primary THA. Lindahl et al. found a probability of 0.6% for late traumatic PPFF, whereas Schwartz et al. reported rates ranging from 4.1% to 28% (half were diagnosed intraoperatively) after uncemented hip replacement, compared with less than 3% when cemented stems were used [6-8].

High morbidity and mortality rates are observed in patients with PPFF. Mortality rates start at 9% at 90-day follow-up, 21% at one-year follow-up, and might increase to a total of 60% at five-year follow-up [9]. Immobility among older (in)patients is related to higher mortality rates [10]. Whereas early postoperative mobilization of surgically treated patients with a variety of medical diagnoses and surgical conditions may improve patient outcomes. [11]. In the literature, different postoperative load-bearing protocols are described [12]. Current practice in patients with surgically treated PPFF mostly includes a postoperative period of non-, restricted-, or partial load bearing for 6-8 weeks [12]. This generally leads to a loss of mobilization and independence and, subsequently, a prolonged length of hospital stay with increased costs of healthcare services [13].

Permissive weight bearing (PWB) has been designed as a new aftercare mobilization regimen to optimize rapid clinical recovery and restoration of functionality [14]. PWB has been proven to be successful (in cases of non-union, wound infection, early removal of implant, implant fracture, and secondary dislocation) in different kinds of lower extremity fractures (pelvic/acetabular, distal femur, tibial plateau, distal tibia/ankle, and foot) without raising post-operative complications when compared to non-load-bearing protocols [14]. In addition, it ensures patients fully mobilize four weeks earlier (12 weeks versus 16 weeks) compared to AO guidelines [14]. Also, in the case of uncemented THA, early postoperative load bearing is proven to be safe and does not increase the incidence of postoperative complications [15].

To the best of our knowledge, no postoperative clinical guidelines after PPFF exist. Therefore, the question was postulated on how orthopedic surgeons deal with postoperative load bearing in the management of surgically treated PPFF. The first aim is to investigate the current postoperative practice of weightbearing instructions for patients with surgically treated PPFF (Vancouver A, B, and C [16]), accounting for differences in types of periprosthetic fractures and treatment options among Dutch orthopedic surgeons. The second aim was to determine whether PWB was already applied in the Netherlands and, if so, how often and for which types of PPFF (Vancouver A, B, and C [16]).

This article was previously presented as a meeting abstract at the 22nd European Congress of Trauma and Emergency Surgery on May 7, 2023, and at the European Federation of National Associations of Orthopedics and Traumatology Congress on May 24, 2023.

## Materials and methods

An online survey was developed (Appendix 1) and distributed among Dutch orthopedic surgeons using online software (Momentive Inc. (formerly SurveyMonkey Inc.), San Mateo, California, United States). This survey contained general questions regarding daily practice, questions on load bearing in postoperative management, and case-specific questions. The survey was presented by e-mail to members of the hip and trauma subgroups, including arthroplasty surgeons, of the Dutch Orthopedic Association on July 19th, and a reminder was sent on August 30th, 2021. The survey was online until September 27th, 2021. 

PWB was defined as described by Kalmet et al. [17]. In their definitions, patients are instructed and trained to start load-bearing as tolerated. The limitation of load bearing is dependent on the patient’s perception of pain, a feeling of instability, and redness or swelling at the fracture site. The primary objective was to quantify the proportion of physicians prescribing certain intensities of load-bearing. For the purpose of secondary analysis (prescription of PWB in high-volume surgeons vs. low-volume surgeons and fellows vs. consultants), a high-volume surgeon was defined as someone who operated more than 10 PPFF annually, and a low-volume surgeon was defined as someone who operated less than 10 PPFF annually.

Statistical analyses were performed using IBM Corp. Released 2020. IBM SPSS Statistics for Windows, Version 27.0. Armonk, NY: IBM Corp. Descriptive statistics were used to describe the demographic data and baseline characteristics. Results are presented as frequencies and percentages (%). A one-way ANOVA test was used to assess a difference in postoperative management between fellows and consultants and between high-volume and low-volume surgeons. 

## Results

Group characteristics 

The survey was sent to 569 orthopedic surgeons, of whom 75 responded (13%). Group characteristics are shown in Table 1. From the respondents, nine (12%) were fellows (sub-specialty training), and 66 (88%) were consultants. Half of all respondents (51%) worked at a non-academic teaching hospital. There was a good distribution among the cohort in terms of experience, ranging from zero to five years to >20 years, and based on the number of interventions regarding PPFF annually. Nearly all respondents (95%) used the Vancouver [16] classification system. The concept of PWB was already known by 61 respondents (81%).

**Table 1:** Group characteristics n = number of respondents, PPFF = periprosthetic femoral fracture, PWB = permissive weight bearing, UCS = Unified Classification System.

Total n (%)	75 (100)
Hospital n (%)	
Academic hospital	8 (11)
Non-academic teaching hospital	38 (51)
Non-academic non-teaching hospital	28 (37)
Independent treatment center	1 (1)
Level of expertise n (%)	
Fellow	9 (12)
Consultant	66 (88)
Number of years working n (%)	
0-5	21 (28)
5-10	22 (29)
10-15	13 (17)
15-20	15 (20)
>20	4 (5)
Number of interventions regarding PPFF annually n (%)	
0-3	14 (19)
4-6	14 (19)
7-9	16 (21)
10-13	18 (24)
14-15	5 (7)
>15	8 (11)
Use of classification system n (%)	
Vancouver	71 (95)
Baba	3 (4)
UCS	1 (1)
Use of standard protocol PPFF n (%)	
Yes	18 (24)
No	57 (76)
Familiar with PWB n (%)	
Yes	61 (81)
No	14 (19)

Postoperative load bearing

**Figure 1:**
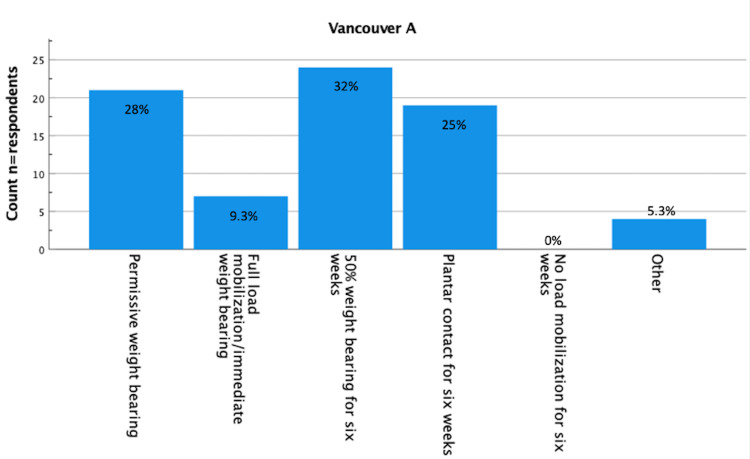
Vancouver A (n=75) You perform a trochanter refixation for a Vancouver A periprosthetic fracture. The fracture is non-comminuted, and with osteosynthesis, there is good reposition and a stable fixation. What is your policy regarding postoperative loading of the operated leg? (Assuming a well-instructed patient). n = number of respondents.

**Figure 2:**
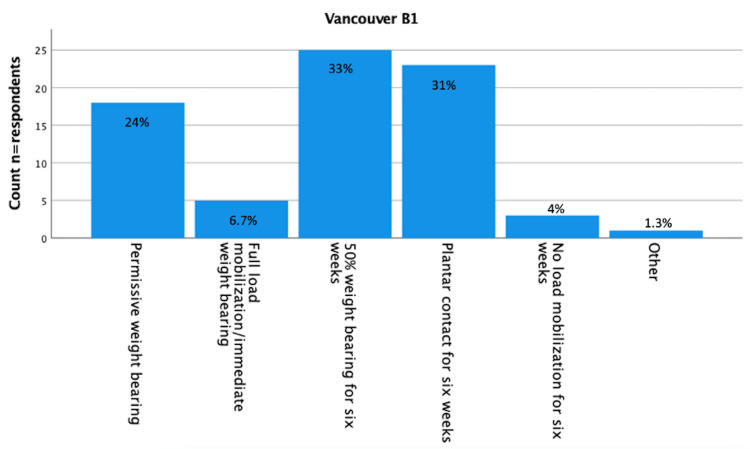
Vancouver B1 (n=75) You are performing plate screw osteosynthesis on a Vancouver B1 (stem-fixed) periprosthetic femoral fracture. The fracture is non-comminuted, and in osteosynthesis, there is good reposition and a stable fixation. What is your policy regarding postoperative loading of the operated leg? (Assuming a well-instructed patient). n = number of respondents.

**Figure 3:**
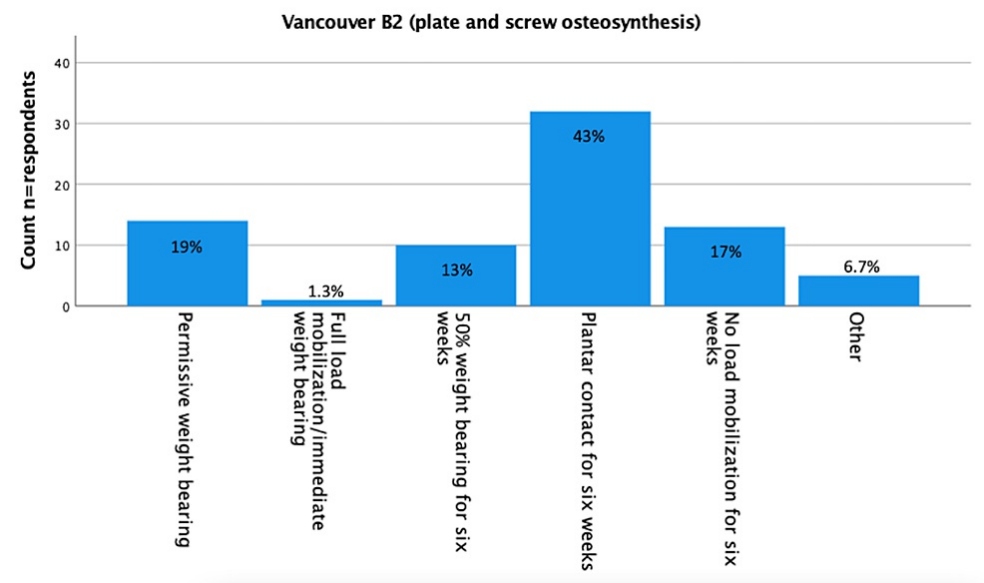
Vancouver B2 (plate/screw osteosynthesis) (n=75) You perform a plate screw osteosynthesis on a Vancouver B2 (stem loose) periprosthetic femoral fracture (cemented stem) (conscious choice due to, for example, patient comorbidity). The fracture is non-comminuted, and in osteosynthesis, there is good reposition and a stable fixation. What is your policy regarding postoperative loading of the operated leg? (Assuming a well-instructed patient). n = number of respondents.

**Figure 4:**
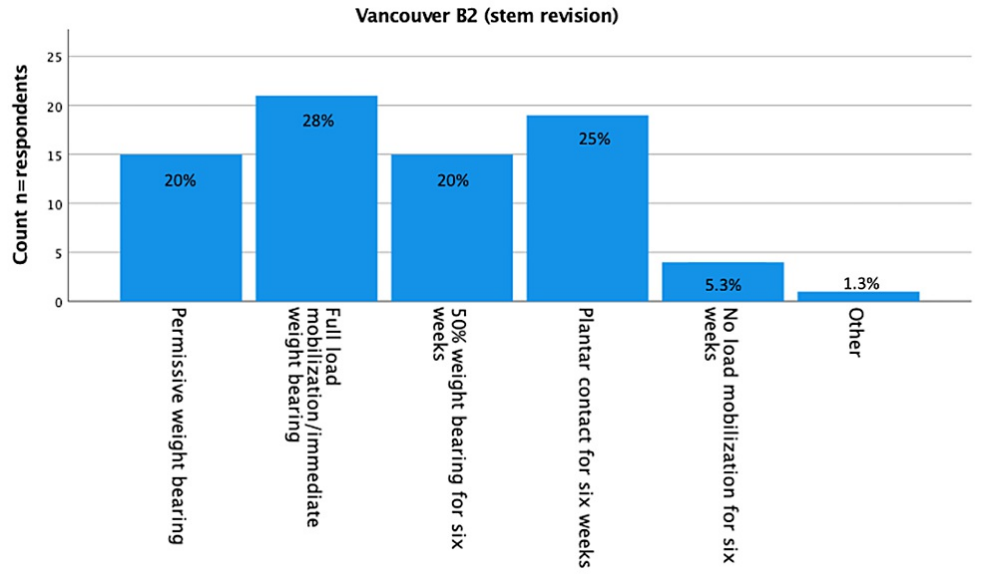
Vancouver B2 (stem revision) (n=75) You perform a stem revision in combination with plate screw osteosynthesis/cerclages. The fracture is non-comminuted, and in osteosynthesis, there is good reposition and a stable fixation. What is your policy regarding postoperative loading of the operated leg? (Assuming a well-instructed patient). n = number of respondents.

**Figure 5:**
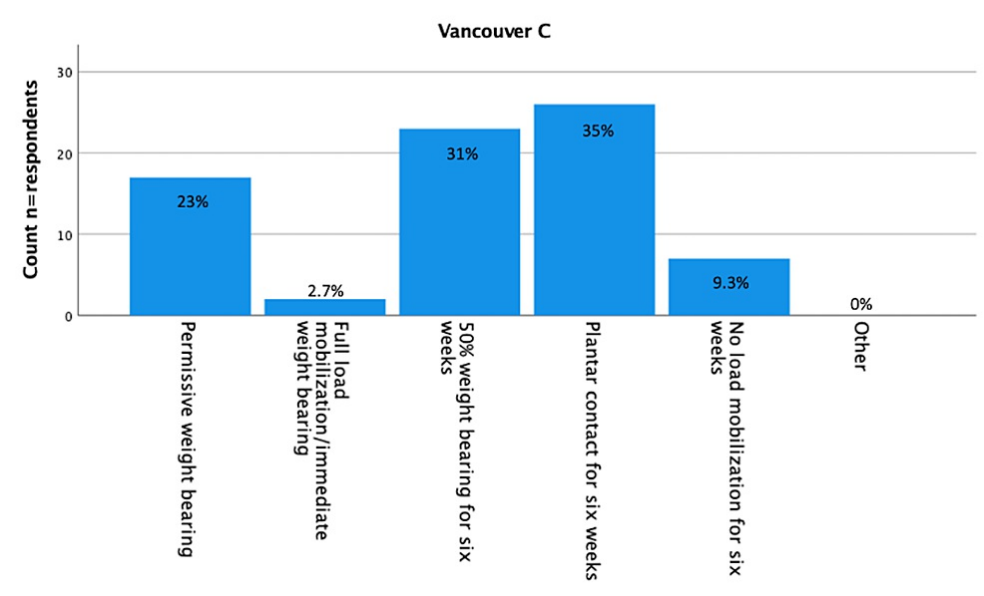
Vancouver C (n=75) You perform plate screw osteosynthesis on a Vancouver C periprosthetic fracture. The fracture is non-comminuted, and in osteosynthesis, there is good reposition and a stable fixation. What is your policy regarding postoperative loading of the operated leg? (Assuming a well-instructed patient). n = number of respondents.

Results about postoperative load bearing after surgically treated Vancouver A (fracture of lesser or greater trochanter [16]), Vancouver B1 (fracture around a well-fixed implant [16]), Vancouver B2 (fracture with a loose implant [16]), and Vancouver C (fracture well below the tip of the implant [16]) are displayed in Figure 1-5. Most respondents (n=24 (32%) for Vancouver A and n=25 (33%) for Vancouver B1) advised 50% load bearing for six weeks. In the case of Vancouver B2 (plate/screw osteosynthesis, conscious choice in view of vulnerable elderly) and Vancouver C, most respondents (n=32 (43%) and 26 (35%) advised plantar contact for six weeks, and for Vancouver B2 (stem revision), most respondents (n=21, 28%) advised full load mobilization or immediate load bearing. PWB was advised by 28% (n=21) for Vancouver A, 24% (n=18) for Vancouver B1, 19% (n=14) for Vancouver B2 (plate/screw osteosynthesis), 20% (n=15) for Vancouver B2 (stem revision), and 23% (n=17) for Vancouver C. 

In all cases, half of the respondents (range 43%-53%, n=32-40) answered that their policy regarding postoperative load bearing of the operated leg would change for a patient who is poorly instructed to mostly no load bearing mobilization for six weeks (range 73%-83%, n=27-33) and to PWB in a few cases (8%-14%, n=3-5). In the case of a poorly instructed patient for Vancouver B1, out of the five respondents who initially advised full load mobilization, two (40%) changed to no load mobilization for six weeks. For Vancouver B2, out of the 21 respondents who initially advised full load mobilization, six (29%) changed to no load mobilization for six weeks. For Vancouver C, out of the two respondents who initially advised full load mobilization, one (50%) changed to no load mobilization for six weeks.

Case specific 

**Figure 6:**
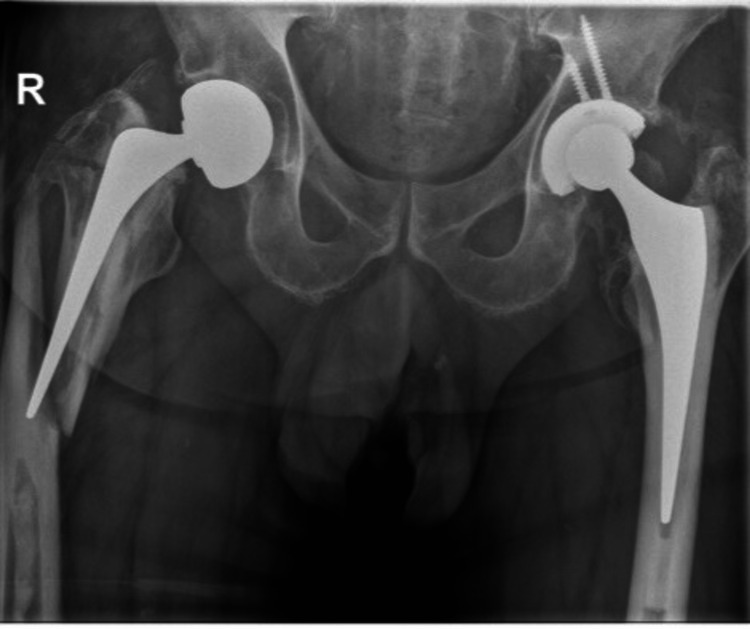
Case A, X-pelvis pre-OR X-ray showing a periprosthetic femoral fracture of the right femur, Vancouver B2 type of fracture

**Figure 7:**
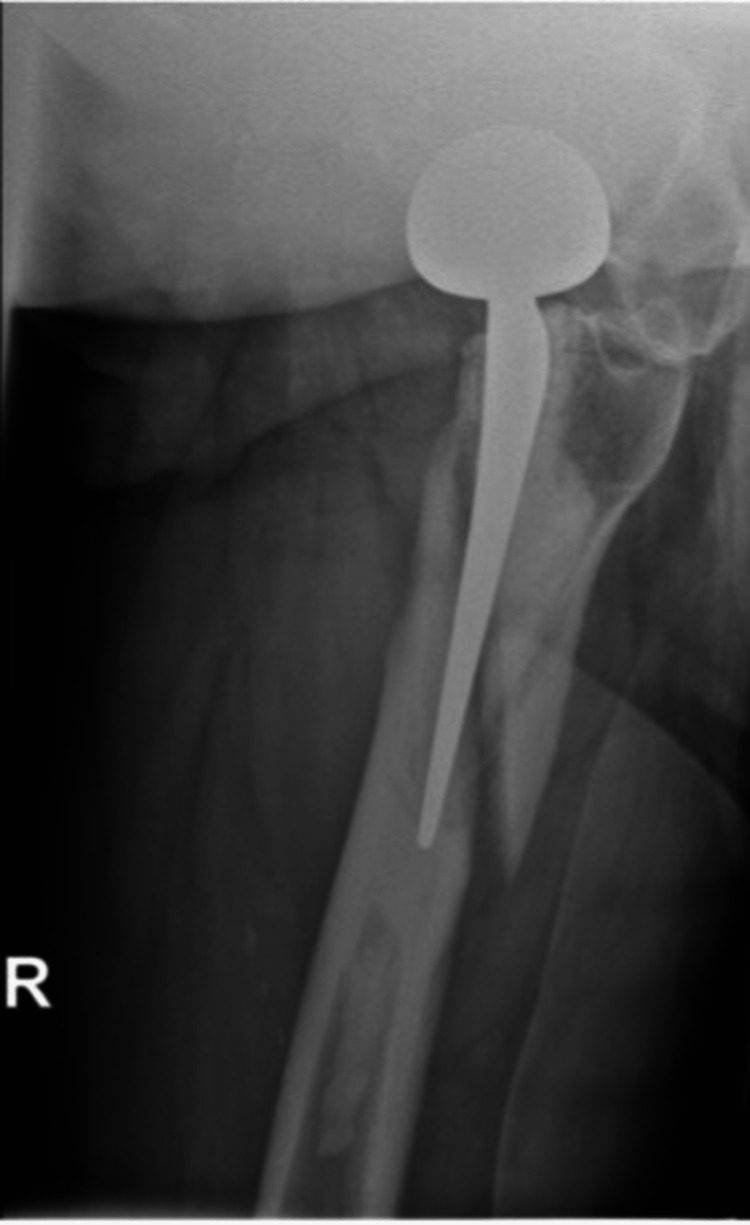
Case A, X-hip-axial pre-OR X-ray showing a periprosthetic femoral fracture of the right femur, Vancouver B2 type of fracture

**Figure 8:**
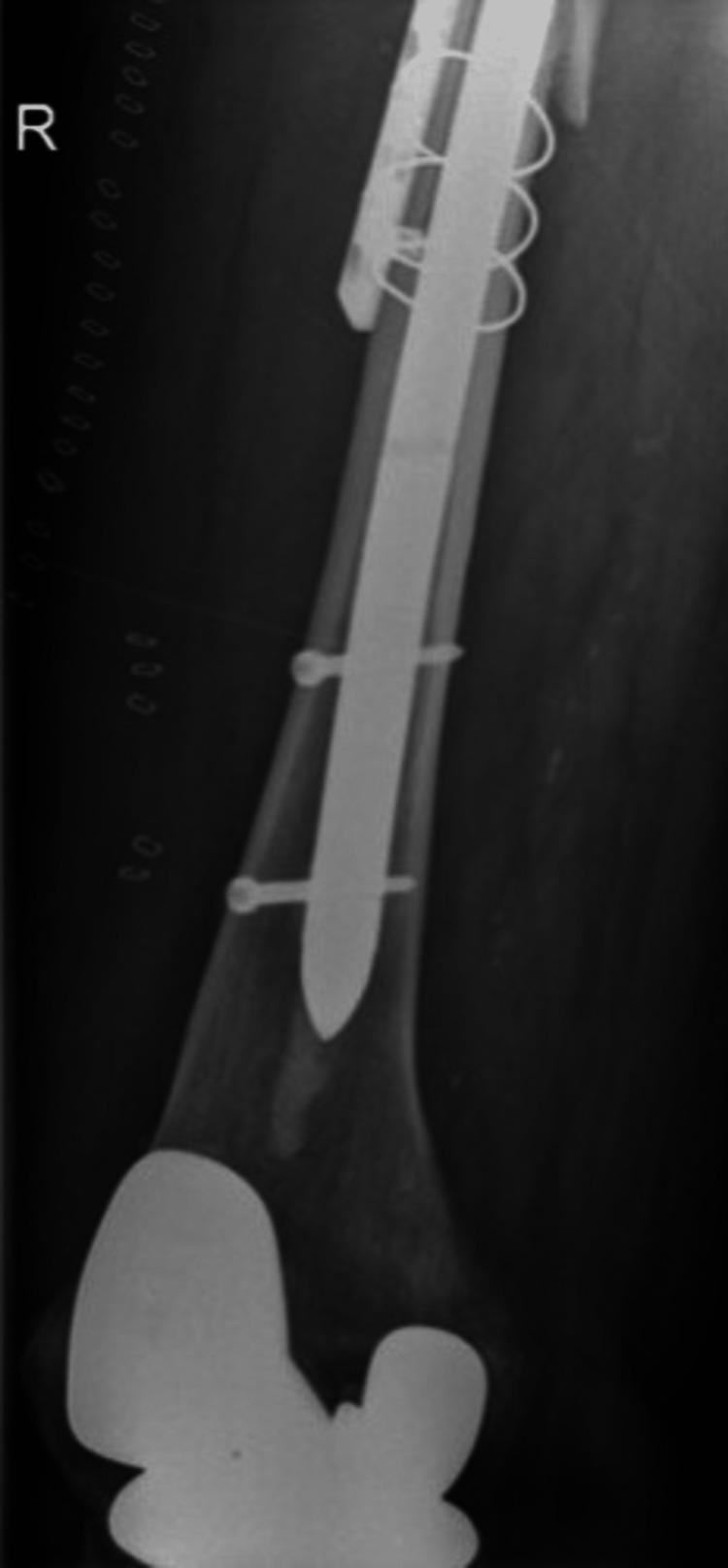
Case A, right X-thigh-axial post-OR The choice was made for a stem revision with an uncemented modular stem and trochanter hook plate with cable grip (and an additional dual-mobility cup)

**Figure 9:**
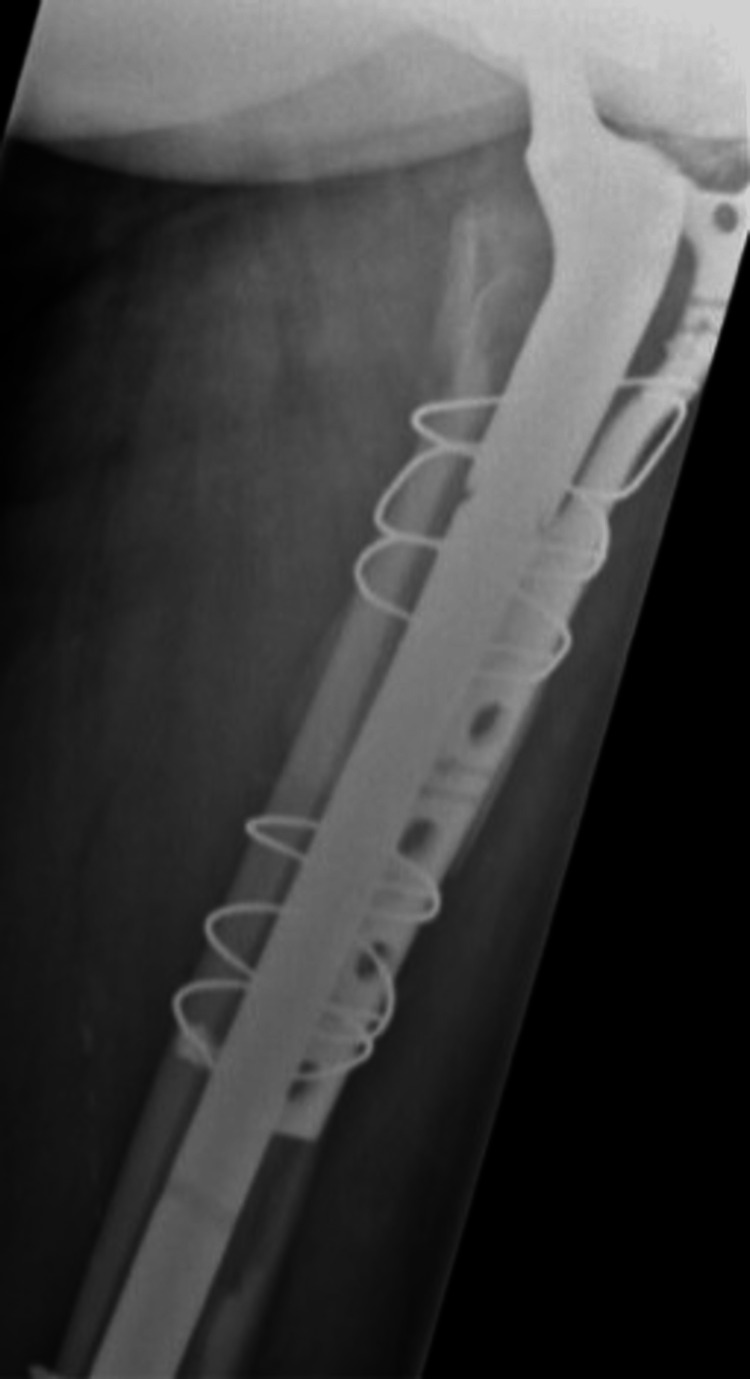
Case A, right X-hip-axial post-OR The choice was made for a stem revision with an uncemented modular stem and trochanter hook plate with cable grip (and an additional dual-mobility cup)

**Figure 10:**
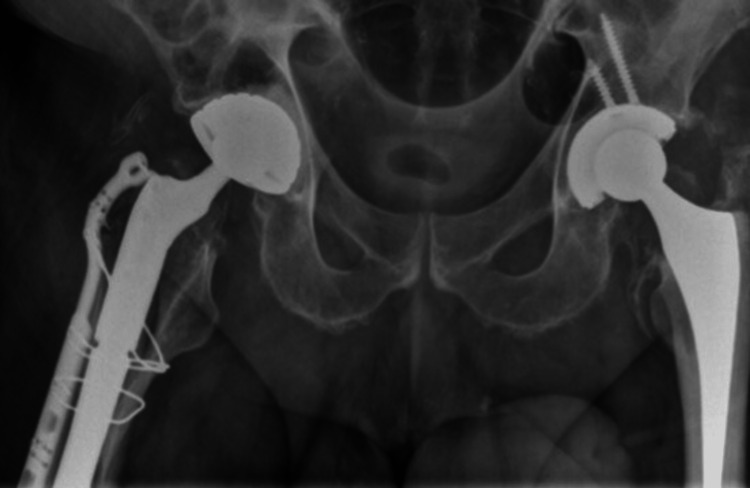
Case A, X-pelvis post-OR The choice was made for a stem revision with an uncemented modular stem and trochanter hook plate with cable grip (and an additional dual-mobility cup)

**Figure 11:**
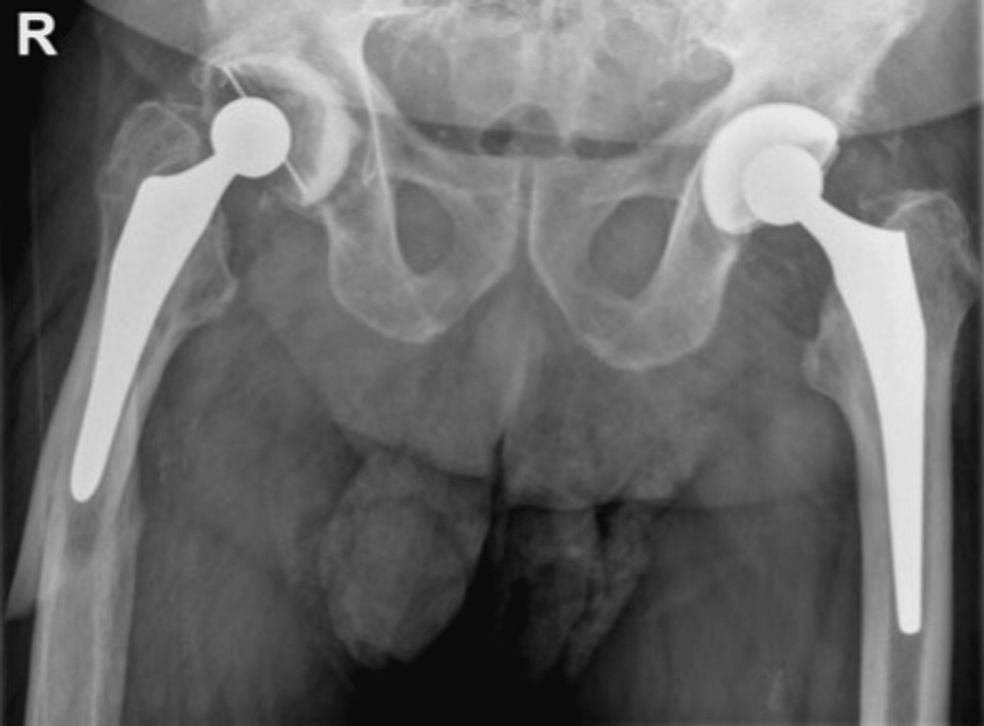
Case B, X-pelvis pre-OR X-ray showing a periprosthetic femoral fracture of the right femur, Vancouver B1 type of fracture

**Figure 12:**
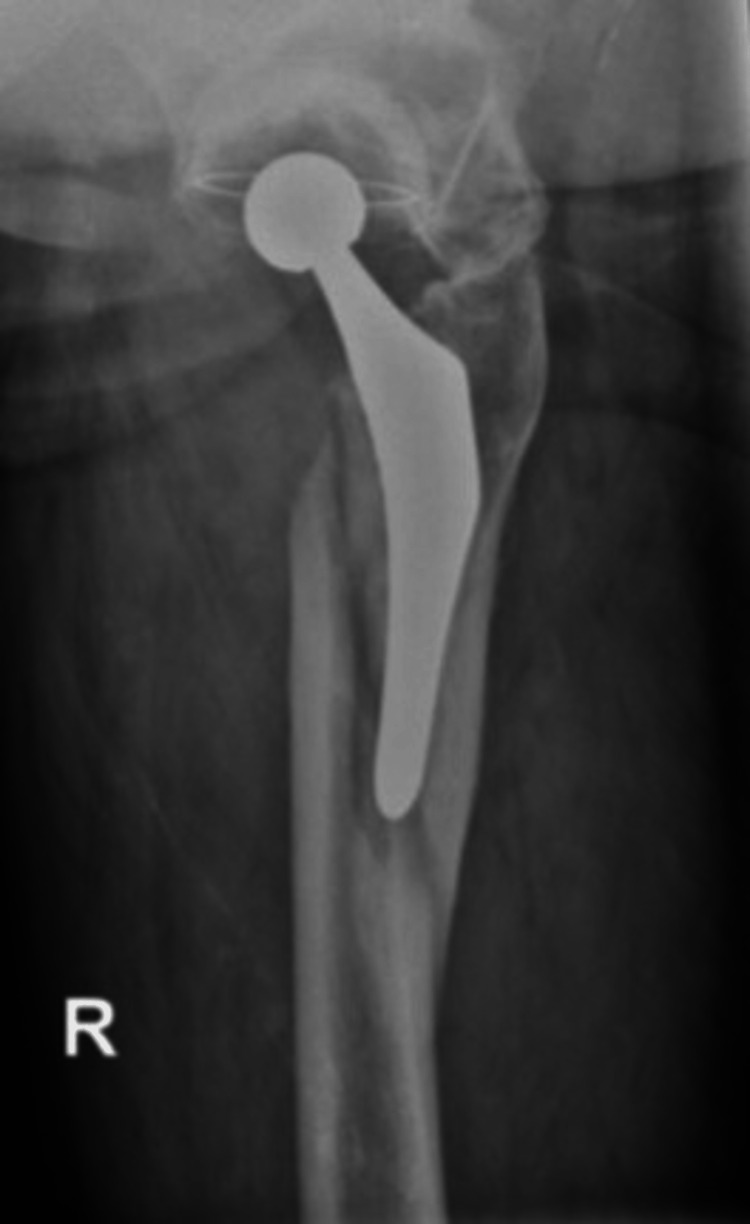
Case B, right X-hip-axial pre-OR X-ray showing a periprosthetic femoral fracture of the right femur, Vancouver B1 type of fracture

**Figure 13:**
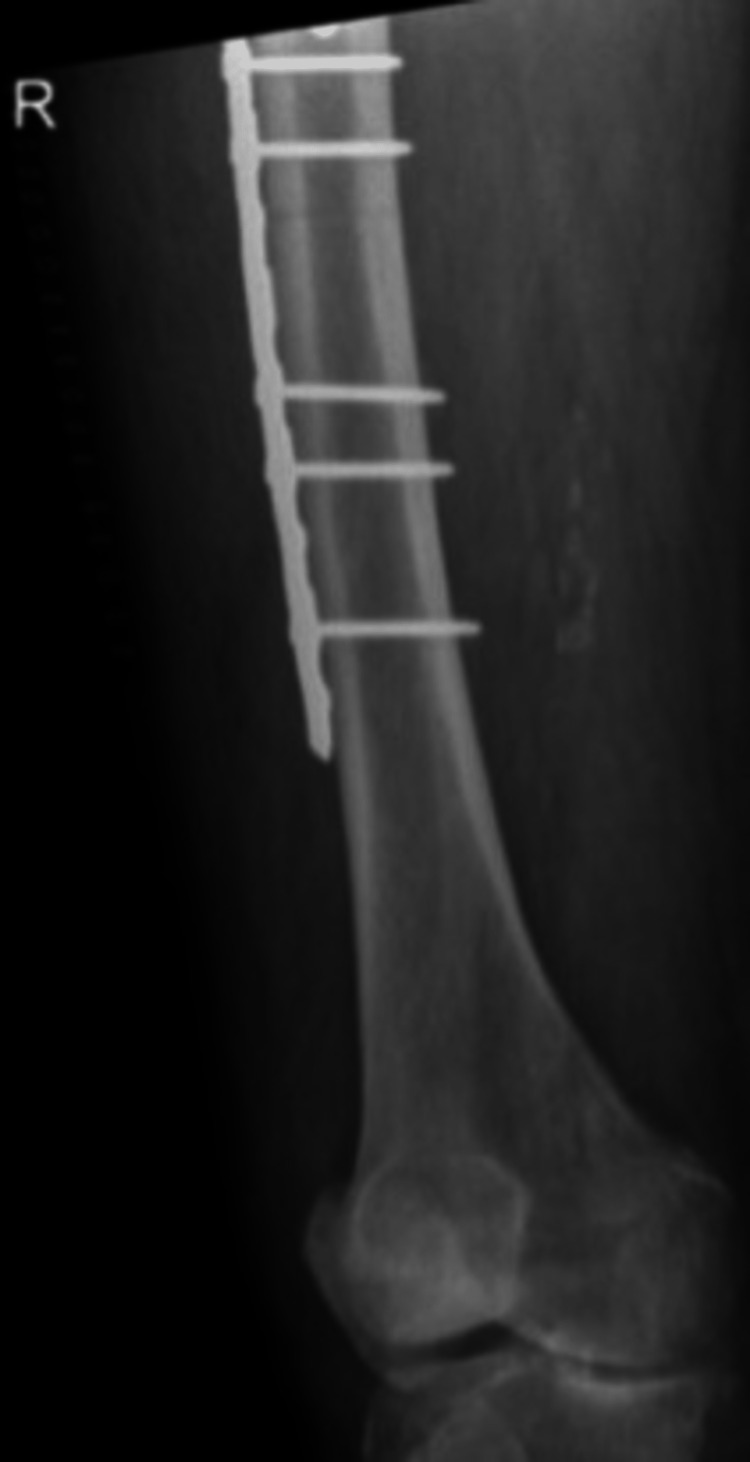
Case B, right X-thigh-AP post-OR The choice was made for plate screw osteosynthesis with the placement of cerclages

**Figure 14:**
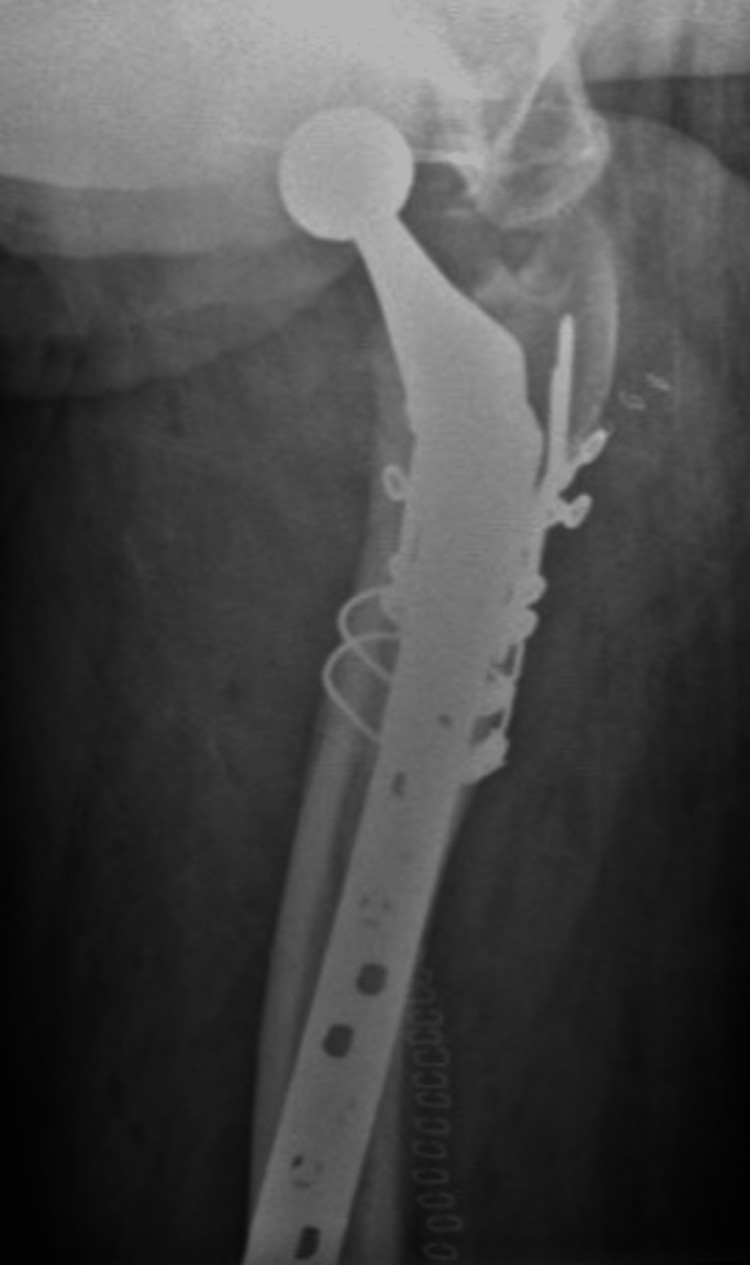
Case B, right X-hip-axial post-OR The choice was made for plate screw osteosynthesis with the placement of cerclages

**Figure 15:**
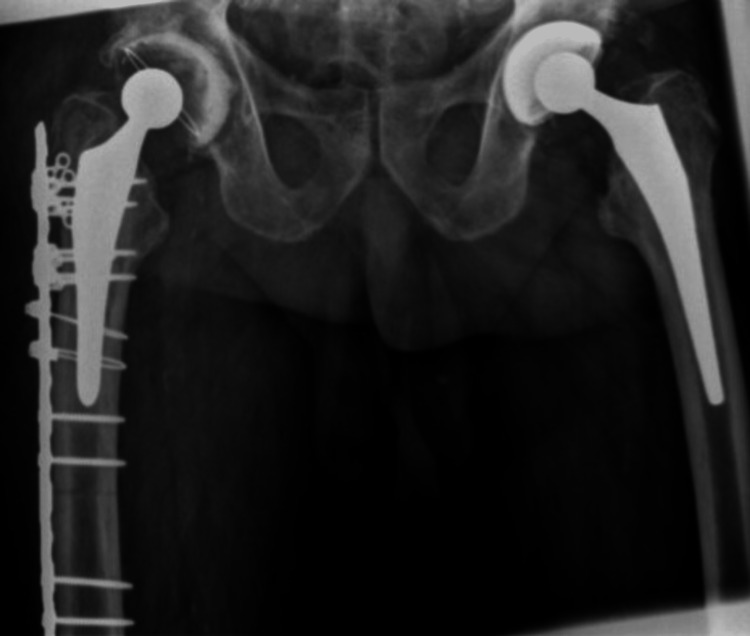
Case B, X-pelvis post-OR The choice was made for plate screw osteosynthesis with the placement of cerclages

**Figure 16:**
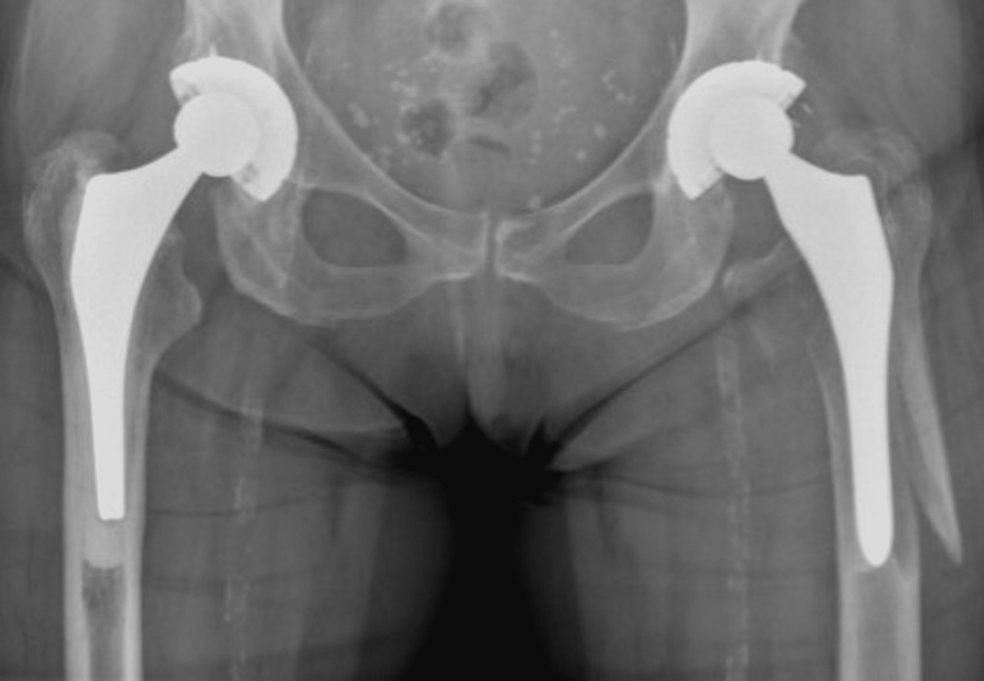
Case C, X-pelvis pre-OR X-ray showing a periprosthetic femoral fracture of the left femur, Vancouver B2 type of fracture

**Figure 17:**
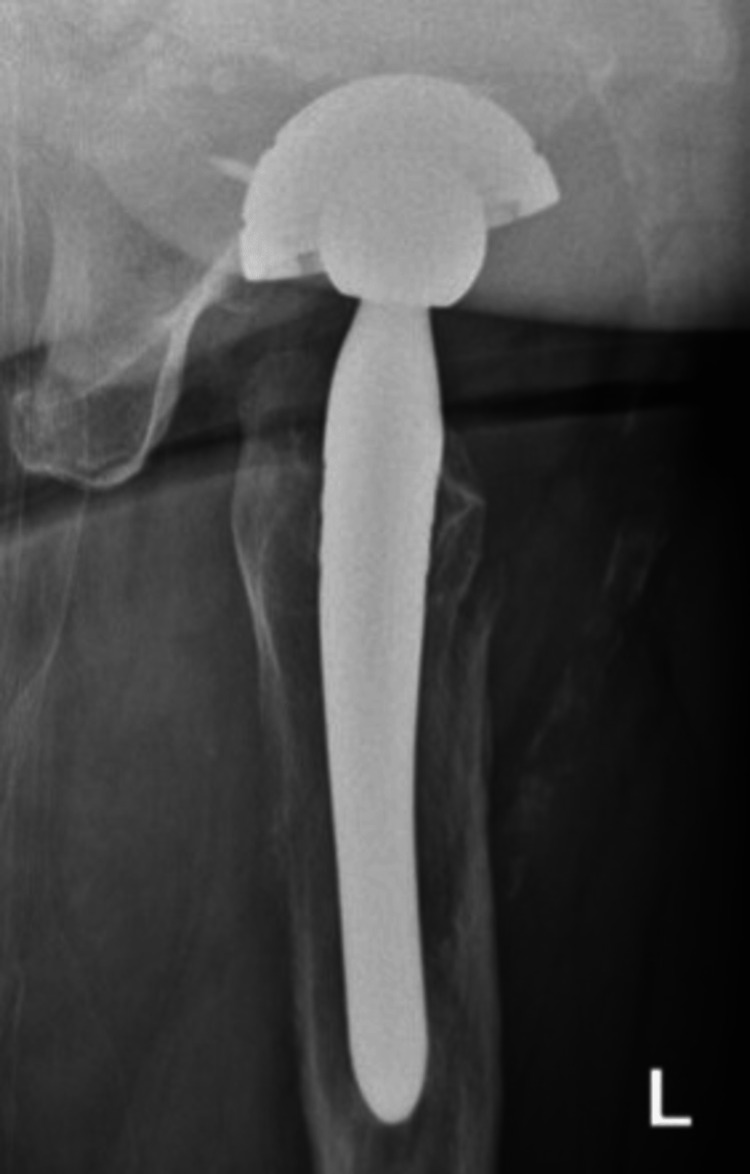
Case C, left X-hip-axial pre-OR X-ray showing a periprosthetic femoral fracture of the left femur, Vancouver B2 type of fracture

**Figure 18:**
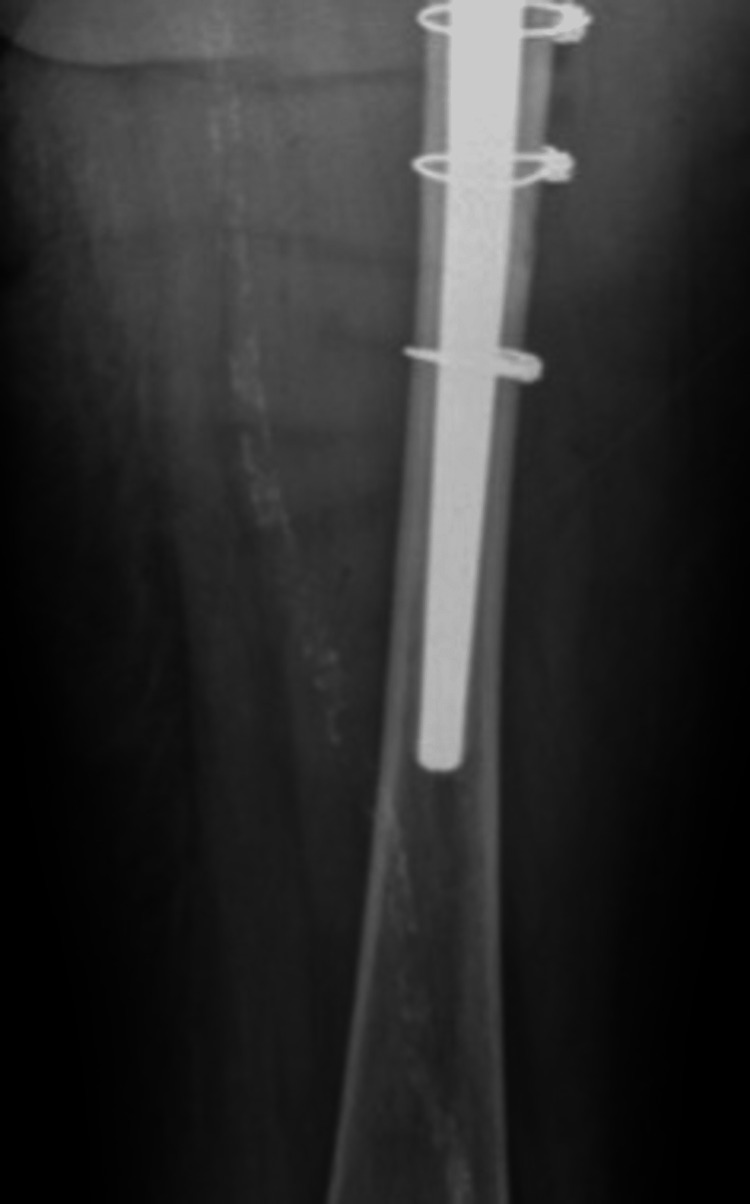
Case C, left X-thigh-axial post-OR The choice was made for a stem revision with an uncemented revision stem and the placement of cerclages

**Figure 19:**
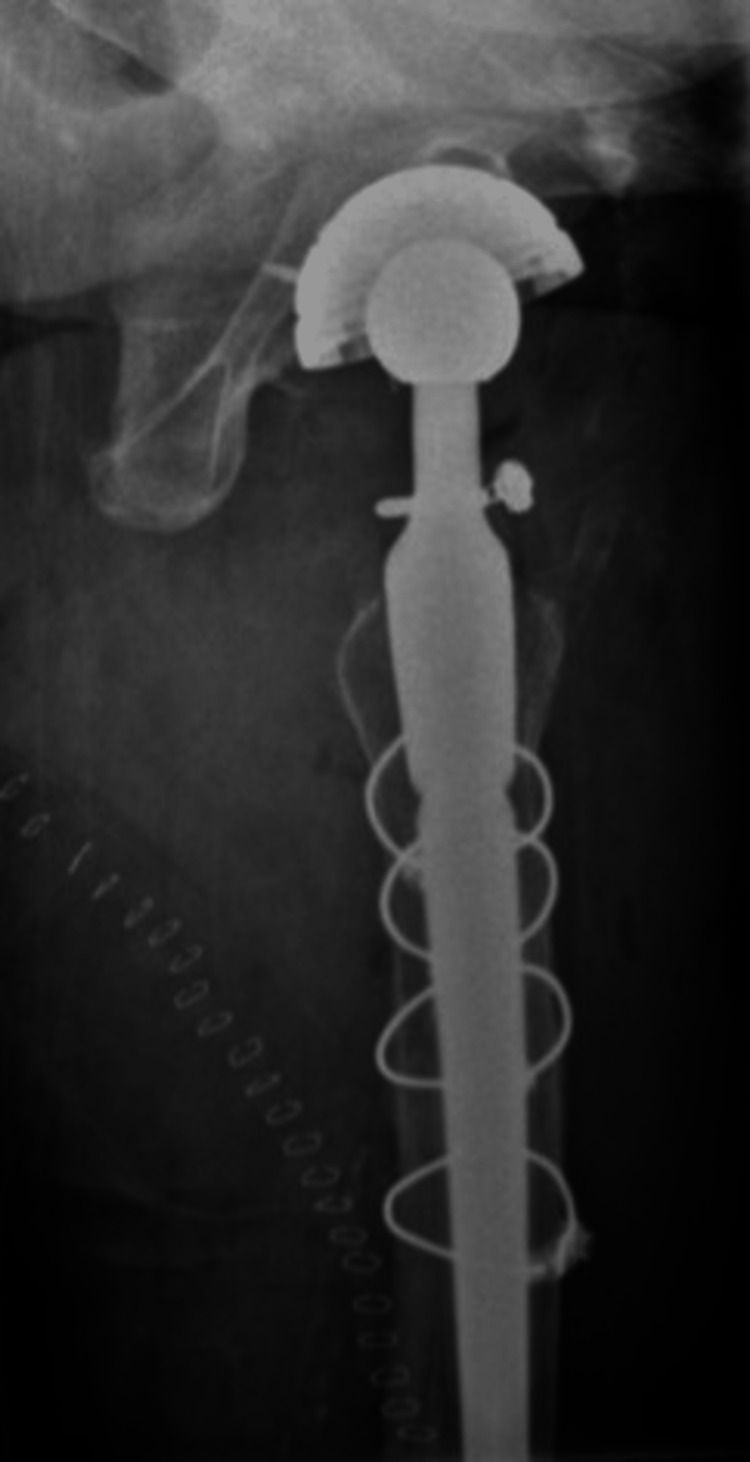
Case C, left X-hip-axial post-OR The choice was made for a stem revision with an uncemented revision stem and the placement of cerclages

**Figure 20:**
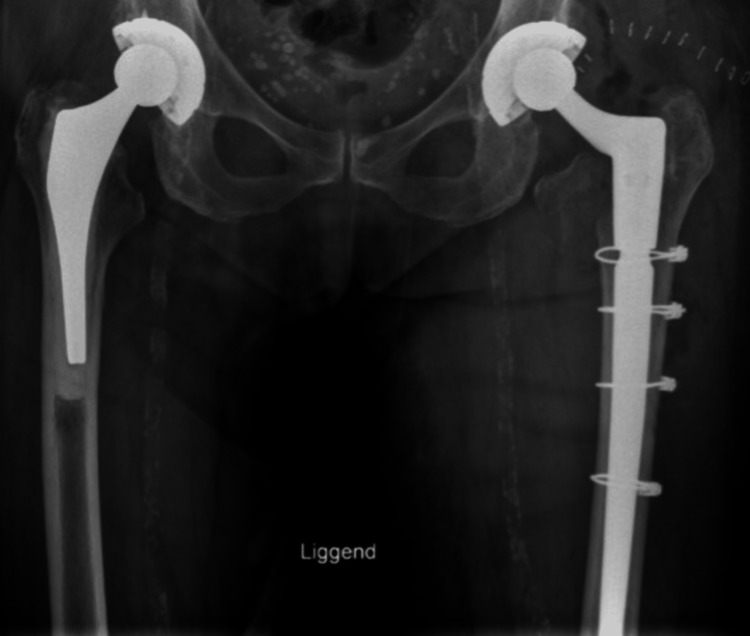
Case C, X-pelvis post-OR The choice was made for a stem revision with an uncemented revision stem and the placement of cerclages

In the case of a stem revision with an uncemented modular stem and trochanteric hook plate with cable grip and additional dual-mobility cup (Case A; Figures 6-10) most respondents (n=23, 32%) advised PWB (Table 2). In the case of plate and screw osteosynthesis and placement of cerclages after the femoral stem was determined to be well fixed (Case B; Figures 11-15), most respondents (n=32, 45%) advised plantar contact for six weeks, and 18 (25%) respondents advised PWB (Table 2). In the case of a stem revision with an uncemented revision stem and placement of cerclages (Case C; Figures 16-20), most respondents (n=25, 35%) advised PWB (Table 2).

**Table 2:** Case specific reactions (n=71) n = number of respondents, PWB = permissive weight bearing.

	Case A	Case B	Case C
Permissive weight bearing (PWB)	n=23, 32%	n=18, 25%	n=25, 35%
Full load mobilization/immediate load bearing	n=11, 16%	n=1, 1%	n=16, 23%
50% load bearing for six weeks	n=19, 27%	n=15, 21%	n=19, 27%
Plantar contact for six weeks	n=15, 21%	n=32, 45%	n=7, 10%
No load mobilization for six weeks	n=3, 4.2%	n=5, 7%	n=4, 5.6%
Other	n=0, 0%	n=0, 0%	n=0, 0%

Differences in postoperative load-bearing management

Fellows (n=5, 56%) used PWB significantly more often than consultants (n=12, 18%) in postoperative load bearing for Vancouver C PPFF (P=0.04). High-volume surgeons (n=7, 23%) prescribed significantly (P<0.05) more PWB for Vancouver B2 (stem revision) than low-volume surgeons (n=7, 15%). 

## Discussion

The main finding of the present study was that postoperative load-bearing regimes differed greatly among Dutch orthopedic surgeons. Additionally, there are no decisive guidelines or consensus in the postoperative load-bearing regime after surgically treated PPFF is used in the Netherlands. Despite a good reposition and stable fixation in well-instructible patients and with non-comminuted fractures obtained with surgery in Vancouver A, B, and C PPFF, PWB was never the first choice in the postoperative load-bearing regime. In contrast, in cases of stem revision (cases A and C), PWB was the first choice as the postoperative load-bearing regime. In the case of Vancouver B2 (plate/screw osteosynthesis, conscious choice in view of the vulnerable elderly), five respondents (6.7%) would not consider performing only open reposition and internal fixation in cases of this type of fracture. However, there is no evidence in the current literature to restrict patients to postoperative load bearing in the case of PPFF. 

Only a few studies have been published about PWB in lower extremity fractures. In their retrospective cohort study, Kalmet et al. concluded that PWB is a safe postoperative load-bearing regime after surgically treated tibial plateau fractures with regards to complication rates [18]. In the PWB group, one non-union and one superficial wound infection were observed versus three non-unions, two superficial wound infections, and one deep infection in the restricted load-bearing group (6.5% vs. 10%). The time to full load bearing was significantly shorter with PWB than with restricted load bearing (15 weeks vs. 21 weeks). PWB was related to reduced time to full load bearing with no differences in quality of life or pain [18]. PWB has also been proven to be successful in other kinds of lower extremity fractures (pelvic/acetabular, distal femur, tibial plateau, distal tibia/ankle, and foot). Meys et al. showed that PWB, despite being the more aggressive rehabilitation regimen, does not lead to increased post-operative complications (non-union, wound infection, early removal of implant, implant fracture, secondary dislocation) when compared to non-load bearing protocols, and it ensures patients have earlier full load bearing mobilization compared to the AO guidelines (12 weeks vs. 16 weeks) [14]. Cunningham et al. conclude in their study that immediate postoperative load bearing as tolerated in patients with intramedullary fixation for subtrochanteric fractures results in reduced length of hospital stay (7.4 days vs. 9.7 days) with no significant differences in re-operations (in case of infection, hardware failure, non-union, or malunion) and complications (11% vs. 11% in non-load bearing) [19]. Also, Lieder et al. found similar results in surgically treated patients with extra-articular distal femoral fractures with similar rates of early adverse events requiring reoperation (11% in load bearing as tolerated vs. 19% in toe touch load bearing) [20]. In addition, no differences between length of stay, malunion, or patient-reported outcomes were observed [20]. These findings are in line with expectations of PWB in the postoperative load-bearing regime for PPFF with good postoperative reposition and stable fixation, given the fact that early mobilization in surgically treated patients leads to improvements in patient outcomes in terms of complications, morbidity, and mortality [11]. In conclusion, the literature clarifies the safety and effectiveness of PWB for lower extremity fractures. 

In literature about load bearing after THA, early load bearing is also proven to be safe. Tian et al. performed a meta-analysis looking at partial load bearing versus early full load bearing [15]. No significant differences were found in postoperative complications (prosthetic loosening, femoral subsidence, radiolucent lines). They concluded that early load mobilization after uncemented THA is safe and does not increase the incidence of postoperative complications. 

Thaler et al. also performed an online survey study to investigate the current treatment of PPFF by members of the European Hip Society (EHS) [21]. They first looked at treatment differences in cases of different PPFF. Secondary, they looked at load-bearing restrictions after surgically treated PPFF. They found strong variations regarding postoperative load-bearing protocols for all Vancouver PPFF. This is also in line with our results. This endorses the need for more standardized protocols for the management of PPFF. 

Still literature lacks studies focusing on postoperative load bearing in general after PPFF. But, given the fact that it is proven to be safe and effective in different kinds of lower extremity fractures, it is likely that this will also be the case in PPFF. Therefore, more research is needed to confirm this hypothesis [22]. 

In secondary analysis, fellows were keener to prescribe PWB as a postoperative load-bearing regime for Vancouver C PPFF. This might be explained by the fact that PWB is a fairly new concept and has only been described as a protocol for lower extremity fractures since 2019 [14]. Hypothetically, fellows may be more familiar with PWB. High-volume surgeons prescribed more PWB compared to low-volume surgeons. This can be due to the fact that high-volume surgeons are more experienced with PPFF management and are, therefore, less reluctant to prescribe PWB as a postoperative load-bearing regime. This study shows that there is currently a lot of variability in postoperative load-bearing regimes. There is a lack of knowledge about the optimal load-bearing treatment after PPFF, and dissemination of this knowledge is slow (results regarding fellows vs. consultants). Concluding PWB can possibly solve two great problems: one is that it is a more effective postoperative treatment option, resulting in less physiological decline in vulnerable patients without increasing complications, and the second is that it will lead to uniformity in postoperative treatment strategy, which is essential for good outcome measurements. 

The main limitation of this study was the response rate of 13%. With 23 questions, the survey took up to 15 minutes to complete, which could have led to an unwillingness to participate. We believe the extensive analysis and questionnaire were necessary to address all aspects of PWB and postoperative management of PPFF. The use of case-specific questions, including pre- and postoperative X-rays, mimicked clinical practice as closely as possible. Nevertheless, there was a homogenous distribution of respondents based on the type of hospital in which they were working, the number of interventions regarding PPFF annually performed, and their years of experience as orthopedic surgeons. For this reason, we believe this study is useful as a baseline with regards to load bearing in postoperative settings of surgically treated PPFF in the Netherlands. Therefore, the results may logically apply mainly to the situation in the Netherlands and are not necessarily extrapolated to other parts of the world. 

## Conclusions

With this study, we intended to give an overview of the current postoperative practice of weight-bearing instructions for patients with surgically treated PPFF, accounting for differences in types of periprosthetic fractures and treatment options and whether PWB was already applied in the Netherlands among Dutch orthopedic surgeons. The present study shows that postoperative load-bearing regimes after surgically treated patients suffering PPFF (Vancouver A, B, and C) differed greatly among Dutch orthopedic surgeons. In the absence of decisive guidelines or consensus, there is a great need for scientific evidence and research on this topic. We would recommend further research to explore the effectiveness of early postoperative mobilization, possibly with PWB, as a postoperative load-bearing protocol in surgically treated PPFF.

## Appendices

Questionnaire: part 1: general questions

1. To which department do you belong? 

a. Orthopedics

b. Trauma surgery

2. Is there a joint transfer/indication discussion/distribution of specific injuries (such as the Multidisciplinary Trauma Unit; MDTU) between the orthopedic department and the trauma surgery department in your center? 

a. Yes

b. No

3. What is the percentage distribution of trauma between orthopedics and trauma surgery in your center? 

4. What kind of hospital do you work at? 

a. University hospital

b. Non-academic teaching hospital (training for orthopedics and traumatology)

c. Non-academic non-teaching hospital

d. Independent treatment center

5. What is your level of expertise? 

a. Fellow

b. Consultant

6. How many years have you been working as a medical specialist? 

a. 0-5

b. 5-10

c. 10-15

d. 15-20

e. >20

7. How many procedures for periprosthetic hip fractures do you perform on an annual basis? 

a. 0-3

b. 4-6

c. 7-9

d. 10-13

e. 13-15

f. >15

8. What surgical techniques do you master to treat periprosthetic hip fractures? 

a. Stem revision

b. Plate screw osteosynthesis (with/without cerclages)

c. Bridging nail

d. Cerclages

e. Conversion to total hip replacement

f. All of the above

9. Which classification system for periprosthetic hip fractures do you routinely use in practice? 

a. Vancouver

b. Baba

c. Unified Classification System

10. Does your center have a standard protocol for perioperative care for patients with periprosthetic hip fractures? 

a. Yes

b. No

11. Are periprosthetic hip fractures treated by a select club within your department?

a. Yes

b. No

12. If yes to question 11, by whom will the periprosthetic hip fractures be handled?

a. Trauma surgeon

b. Orthopedic surgeon with a focus on hip prosthesis

c. Orthopedic traumatologist

13. Are you familiar with the principle of permissive weight bearing (PWB)?

a. Yes

b. No

Part 2: specific questions regarding load bearing in postoperative management

14. You perform a trochanter refixation for a Vancouver A periprosthetic fracture. The fracture is non-comminuted, and with osteosynthesis there is good reposition and a stable fixation. What is your policy regarding postoperative loading of the operated leg? (Assuming a well-instructed patient). 

a. Mobilize without load for 6 weeks

b. Touching/plantar contact (10%) for 6 weeks

c. 50% load bearing for 6 weeks

d. Full load mobilization/immediate load bearing

e. Permissive weight bearing in accordance with the mentioned definition 

f. Other, namely: 

15. You are performing a plate screw osteosynthesis on a Vancouver B1 (stem fixed) periprosthetic femoral fracture. The fracture is non-comminuted, and in osteosynthesis, there is good reposition and a stable fixation. What is your policy regarding postoperative loading of the operated leg? (Assuming a well-instructed patient).

a. Mobilize without load for 6 weeks

b. Touching/plantar contact (10%) for 6 weeks

c. 50% load bearing for 6 weeks

d. Full load mobilization/immediate load bearing

e. Permissive weight bearing in accordance with the mentioned definition 

f. Other, namely: 

16. You perform a plate screw osteosynthesis on a Vancouver B2 (stem loose) periprosthetic femoral fracture (cemented stem) (conscious choice due to, for example, patient comorbidity). The fracture is non-comminuted, and in osteosynthesis, there is good reposition and a stable fixation. What is your policy regarding postoperative loading of the operated leg? (Assuming a well-instructed patient).

a. Mobilize without load for 6 weeks

b. Touching/plantar contact (10%) for 6 weeks

c. 50% load bearing for 6 weeks

d. Full load mobilization/immediate load bearing

e. Permissive weight bearing in accordance with the mentioned definition 

f. Other, namely: 

17. You perform a stem revision in combination with plate screw osteosynthesis/cerclages. The fracture is non-comminuted, and in osteosynthesis, there is good reposition and a stable fixation. What is your policy regarding postoperative loading of the operated leg? (Assuming a well-instructed patient).

a. Mobilize without load for 6 weeks

b. Touching/plantar contact (10%) for 6 weeks

c. 50% load bearing for 6 weeks

d. Full load mobilization/immediate load bearing

e. Permissive weight bearing in accordance with the mentioned definition 

f. Other, namely: 

18. You perform a plate screw osteosynthesis on a Vancouver C periprosthetic fracture. The fracture is non-comminuted, and in osteosynthesis, there is good reposition and a stable fixation. What is your policy regarding postoperative loading of the operated leg? (Assuming a well-instructed patient).

a. Mobilize without load for 6 weeks

b. Touching/plantar contact (10%) for 6 weeks

c. 50% load bearing for 6 weeks

d. Full load mobilization/immediate load bearing

e. Permissive weight bearing in accordance with the mentioned definition 

f. Other, namely: 

19. Does the degree of consolidation determine the time of full load bearing? 

a. Yes

b. No

20. Previous questions relate to patients who can be instructed properly. Do you change your postoperative loading protocol for patients who are poorly instructible?

a. Yes/No

b. If yes, how

i. In connection with Vancouver A:

ii. In connection with Vancouver B1:

iii. In connection with Vancouver B2/3:

iv. In connection with Vancouver C:

Part 3: case-specific questions

21. View Figures 1-5. The choice has been made for a stem revision with an uncemented modular stem and trochanter hook plate with cable grip (and additional dual-mobility cup). In view of the above intervention and treatment, what would your policy be regarding postoperative loading? (assuming a well-instructed patient)

a. Mobilize without load for 6 weeks

b. Touching/plantar contact (10%) for 6 weeks

c. 50% load-bearing for 6 weeks

d. Full load mobilization/immediate load bearing

e. Permissive weight bearing in accordance with the mentioned definition 

f. Other, namely: 

22. View Figures 6-10. The stem was fixed per operatively, a plate screw osteosynthesis was chosen with the placement of cerclages. In view of the above intervention and treatment, what would your policy be regarding postoperative loading? (assuming a well-instructed patient)

a. Mobilize without load for 6 weeks

b. Touching/plantar contact (10%) for 6 weeks

c. 50% load-bearing for 6 weeks

d. Full load mobilization/immediate load bearing

e. Permissive weight bearing in accordance with the mentioned definition 

f. Other, namely: 

23. View Figures 11-15. A stem revision with an uncemented revision stem and placement of cerclages has been chosen. In view of the above intervention and treatment, what would your policy be regarding postoperative loading?

a. Mobilize without load for 6 weeks

b. Touching/plantar contact (10%) for 6 weeks

c. 50% load-bearing for 6 weeks

d. Full load mobilization/immediate load bearing

e. Permissive weight bearing in accordance with the mentioned definition 

f. Other, namely: 

## References

[1] Knight SR, Aujla R, Biswas SP (2011). Total Hip Arthroplasty - over 100 years of operative history. Orthop Rev (Pavia).

[2] HO IB, LE MI (1954). Artificial hip prosthesis in acute and nonunion fractures of the femoral neck: follow-up study of seventy cases. J Am Med Assoc.

[3] Shah RP, Sheth NP, Gray C, Alosh H, Garino JP (2014). Periprosthetic fractures around loose femoral components. J Am Acad Orthop Surg.

[4] Marsland D, Mears SC (2012). A review of periprosthetic femoral fractures associated with total hip arthroplasty. Geriatr Orthop Surg Rehabil.

[5] Della Rocca GJ, Leung KS, Pape HC (2011). Periprosthetic fractures: epidemiology and future projections. J Orthop Trauma.

[6] Lindahl H, Garellick G, Regnér H, Herberts P, Malchau H (2006). Three hundred and twenty-one periprosthetic femoral fractures. J Bone Joint Surg Am.

[7] Schwartz JT Jr, Mayer JG, Engh CA (1989). Femoral fracture during non-cemented total hip arthroplasty. J Bone Joint Surg Am.

[8] Schmidt AH, Kyle RF. (2002). Periprosthetic Fractures of the Femur. Orthopedic Clinics of North America.

[9] Khan T, Middleton R, Alvand A, Manktelow AR, Scammell BE, Ollivere BJ (2020). High mortality following revision hip arthroplasty for periprosthetic femoral fracture. Bone Joint J.

[10] Silva TJ, Jerussalmy CS, Farfel JM, Curiati JA, Jacob-Filho W (2009). Predictors of in-hospital mortality among older patients. Clinics (Sao Paulo).

[11] Pashikanti L, Von Ah D (2012). Impact of early mobilization protocol on the medical-surgical inpatient population: an integrated review of literature. Clin Nurse Spec.

[12] Ricci WM (2015). Periprosthetic femur fractures. J Orthop Trauma.

[13] Lyons RF, Piggott RP, Curtin W, Murphy CG (2018). Periprosthetic hip fractures: A review of the economic burden based on length of stay. J Orthop.

[14] Meys G, Kalmet PH, Sanduleanu S (2019). A protocol for permissive weight-bearing during allied health therapy in surgically treated fractures of the pelvis and lower extremities. J Rehabil Med.

[15] Tian P, Li ZJ, Xu GJ, Sun XL, Ma XL (2017). Partial versus early full weight bearing after uncemented total hip arthroplasty: a meta-analysis. J Orthop Surg Res.

[16] Duncan CP, Masri BA (1995). Fractures of the femur after hip replacement. Instr Course Lect.

[17] Kalmet PH, Meys G, V Horn YY (2018). Permissive weight bearing in trauma patients with fracture of the lower extremities: prospective multicenter comparative cohort study. BMC Surg.

[18] Kalmet PH, Van Horn YY, Sanduleanu S, Seelen HA, Brink PR, Poeze M (2019). Patient-reported quality of life and pain after permissive weight bearing in surgically treated trauma patients with tibial plateau fractures: a retrospective cohort study. Arch Orthop Trauma Surg.

[19] Cunningham BP, Ali A, Parikh HR (2021). Immediate weight bearing as tolerated (WBAT) correlates with a decreased length of stay post intramedullary fixation for subtrochanteric fractures: a multicenter retrospective cohort study. Eur J Orthop Surg Traumatol.

[20] Lieder CM, Gaski GE, Virkus WW, Kempton LB (2021). Is Immediate Weight-Bearing Safe After Single Implant Fixation of Elderly Distal Femur Fractures?. J Orthop Trauma.

[21] Thaler M, Weiss C, Lechner R, Epinette JA, Karachalios TS, Zagra L (2023). Treatment of periprosthetic femoral fractures following total hip arthroplasty: results of an online survey of the European Hip Society. Hip Int.

[22] Asghar A, Kumar A, Kant Narayan R, Naaz S (2020). Is the cortical capillary renamed as the transcortical vessel in diaphyseal vascularity?. Anat Rec (Hoboken).

